# Redox manipulation of the manganese metal in human manganese superoxide dismutase for neutron diffraction

**DOI:** 10.1107/S2053230X18011299

**Published:** 2018-09-21

**Authors:** Jahaun Azadmanesh, William E. Lutz, Kevin L. Weiss, Leighton Coates, Gloria E. O. Borgstahl

**Affiliations:** a Eppley Institute for Research in Cancer and Allied Diseases, 986805 Nebraska Medical Center, Omaha, NE 68198-6805, USA; b Department of Biochemistry and Molecular Biology, 985870 Nebraska Medical Center, Omaha, NE 68198-5870, USA; c Biology and Soft Matter Division, Oak Ridge National Laboratory, 1 Bethel Valley Road, Oak Ridge, TN 37831, USA

**Keywords:** manganese superoxide dismutase, neutron diffraction, perdeuteration, human, oxidation, reduction, large unit cell

## Abstract

Human manganese superoxide dismutase (MnSOD) is one of the most significant enzymes in preventing mitochondrial dysfunction and related diseases by combating reactive oxygen species (ROS) in the mitochondrial matrix. Mitochondria are the source of up to 90% of cellular ROS generation, and MnSOD performs its necessary bioprotective role by converting superoxide into oxygen and hydrogen peroxide. This vital catalytic function is conducted *via* cyclic redox reactions between the substrate and the active-site manganese using proton-coupled electron transfers. Owing to protons being difficult to detect experimentally, the series of proton transfers that compose the catalytic mechanism of MnSOD are unknown. Here, methods are described to discern the proton-based mechanism using chemical treatments to control the redox state of large perdeuterated MnSOD crystals and subsequent neutron diffraction. These methods could be applicable to other crystal systems in which proton information on the molecule in question in specific chemical states is desired.

## Abstract 2

Human mitochondrial manganese superoxide dismutase (MnSOD) is a major player in combating reactive oxygen species in the human body. Methods have been found to control the redox state of the active-site metal of large perdeuterated MnSOD crystals. Neutron diffraction data from these crystals were collected to study the effect of the redox state on proton location. These methods can be applied to other crystal systems where information on the location of protons in specific chemical states is needed.

## 1. Introduction   

Superoxide dismutases (SODs) are essential metalloenzymes that protect against damage caused by reactive oxygen species (ROS) by using an active-site metal to convert superoxide (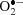) into either oxygen (O_2_) or hydrogen peroxide (H_2_O_2_). The oxidation state of the metal determines whether oxygen or hydrogen peroxide is produced. A trivalent metal oxidizes superoxide to form oxygen, and a divalent metal reduces superoxide to form hydrogen peroxide. The overall enzymatic activity of SODs can thereby consist of cycling oxidation states of the metal to convert superoxide into its products:
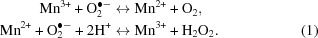



SODs are classified based on whether nickel, iron, copper and zinc, or manganese is coordinated at the active site. Prokaryotes contain all four types of SOD, while eukaryotes contain and compartmentalize three types: FeSOD is found in chloroplasts, CuZnSOD localizes to the cytoplasm and extracellular matrix, and MnSOD dwells within the mitochondria. Of the two SODs found in humans, MnSOD and CuZnSOD, MnSOD is arguably the most vital owing to its localization.

The mitochondria produces up to 90% of cellular ROS from generation of superoxide and its derivatives, such as hydroxyl radicals (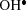), peroxynitrite (ONOO^−^) and nitrogen dioxide (NO_2_) (Gao *et al.*, 2008 ▸; Jastroch *et al.*, 2010 ▸). The electron-transport chain (ETC) of the mitochondria is the source of large amounts of superoxide formation within a cell. Electrons being shuttled across the ETC can leak and perform a one-electron reduction of diatomic oxygen to yield superoxide (Jastroch *et al.*, 2010 ▸). The presence of MnSOD within the matrix safeguards against excessive amounts of superoxide accumulation and ultimately prevents mitochondrial dysfunction from oxidative stress. The capacity of MnSOD to decrease superoxide levels in the mitochondria is associated with longevity and the presence/absence of pathologies (Landis & Tower, 2005 ▸). Fruit flies overexpressing MnSOD have an increased lifespan, whereas MnSOD-knockout mice die within the first 10 d of life owing to significant cardiac and neurological dysfunction (Li *et al.*, 1995 ▸; Sun *et al.*, 2002 ▸). MnSOD polymorphisms are associated with the presence of hypertension, both types of diabetes, prostate cancer and ovarian cancer (Lebovitz *et al.*, 1996 ▸; Nakanishi *et al.*, 2008 ▸; Arsova-Sarafinovska *et al.*, 2008 ▸). These studies suggest that MnSOD has application for therapeutic approaches (Tarhini *et al.*, 2011 ▸; Miriyala *et al.*, 2012 ▸; Borrelli *et al.*, 2014 ▸). The crucial bioprotective role of MnSOD has instigated our experiments, which are designed to understand its catalytic mechanism.

A large body of literature has attempted to discern the catalytic mechanism of human MnSOD, but this still remains enigmatic (Lah *et al.*, 1995 ▸; Whittaker & Whittaker, 1996 ▸; Hearn *et al.*, 1999 ▸, 2003 ▸; Li *et al.*, 1999 ▸; Borgstahl *et al.*, 2000 ▸; Lévêque *et al.*, 2001 ▸; Miller *et al.*, 2003 ▸; Jackson *et al.*, 2004 ▸; Noodleman *et al.*, 2004 ▸; Abreu *et al.*, 2005 ▸; Srnec *et al.*, 2009 ▸; Abreu & Cabelli, 2010 ▸; Porta *et al.*, 2010 ▸; Azadmanesh & Borgstahl, 2018 ▸). The difficulty in elucidating the mechanism is attributed to limitations in detecting hydrogen positions at the active site, especially by X-ray crystallography. Knowledge of the hydrogen positions is paramount to understanding the mechanism because the cyclic redox reaction of MnSOD relies on the coupling of electron transfers to proton transfers (also called proton-coupled electron transfers; PCETs; Maliekal *et al.*, 2002 ▸). A consensus in the literature on MnSOD is that proton-shuttling relays are present around the manganese ion to mediate systematic PCETs, as noted in the detailed reviews by Abreu & Cabelli (2010 ▸) and Azadmanesh & Borgstahl (2018 ▸). This facilitates one of the fastest (*k*_cat_ = 40 000 s^−1^) and most efficient (*k*_cat_/*K*_m_ close to 10^9^ *M* ^−1^ s^−1^) reactions among all enzymes (Guan *et al.*, 1998 ▸). At least two unique relays exist, where the utilization of a relay is based on the oxidation state of the manganese. The exact path of the proton relays has yet to be determined (Srnec *et al.*, 2009 ▸; Heimdal *et al.*, 2011 ▸; Azadmanesh & Borgstahl, 2018 ▸).

Neutron protein crystallography is able to find hydrogen positions, unlike its X-ray counterpart (Golden & Vrielink, 2014 ▸; O’Dell *et al.*, 2016 ▸). This is a consequence of the neutron coherent scattering lengths of D atoms being on par with those of carbon, nitrogen and oxygen, whereas the X-ray scattering lengths of hydrogen and deuterium are approximately an order of magnitude less than those of the second-row organic atoms (Hale *et al.*, 1990 ▸). Deuterium is especially noteworthy for neutron diffraction because it has 40-fold less incoherent scattering (*i.e.* background noise) compared with hydrogen and scatters positively, whereas hydrogen scatters negatively (O’Dell *et al.*, 2016 ▸). Perdeuteration, in which every H atom is replaced by deuterium, takes advantage of the properties of deuterium to improve the neutron diffraction of a sample. Since 50–80% of a protein crystal is typically water, using deuterated water (D_2_O) and as many deuterated mother-liquor components as possible causes a large reduction in incoherent neutron scattering, reducing the background. Half of all atoms found in biological macromolecules are hydrogen; therefore, replacing all H atoms with deuterium in the protein (perdeuteration) gives a sample that is fully optimized for neutron data collection. Perdeuteration also negates the issue of nuclear scattering density cancellation, in which a combination of positive and negative scattering of nearby atoms yields a net scattering of zero. Combining the above use of deuterium and neutron crystallography makes it possible to discern the catalytic mechanism of MnSOD.

Investigating the mechanisms of oxidoreductase metallo­proteins is challenging using X-ray diffraction. Metals are susceptible to being reduced by X-ray radiation; thus studying a completely oxidized metal complex is not feasible (Carugo & Djinović Carugo, 2005 ▸). Even X-ray structures of MnSOD that are published as containing fully oxidized manganese ions are actually partially reduced, as determined by quantum-mechanical and molecular modeling (Li *et al.*, 1999 ▸; Rulíšek & Ryde, 2006 ▸; Heimdal *et al.*, 2011 ▸). When using neutrons, which are a non-ionizing probe, no radiation damage and consequently no redox changes occur to the metals (O’Dell *et al.*, 2016 ▸; Schaffner *et al.*, 2017 ▸). As MnSOD utilizes differing proton relays based on the redox state of the manganese, being able to study both fully oxidized and fully reduced samples is crucial to understanding its mechanism (Srnec *et al.*, 2009 ▸; Heimdal *et al.*, 2011 ▸; Azadmanesh & Borgstahl, 2018 ▸).

In our previous work, we created a crystal system for MnSOD that was reliably applicable to neutron crystallo­graphy (Azadmanesh, Trickel, Weiss *et al.*, 2017 ▸). The known MnSOD crystal system in a high-symmetry space group (*P*6_1_22) was attractive since all of the precipitating agents could be purchased in a deuterated form and complete, redundant data could be collected in a handful of data frames (as neutron beamtime is precious). Unfortunately, the cell dimensions are large (*a* = *b* = 90, *c* = 241 Å); this is the largest unit cell for which hydrogen positions have been visualized to date. Large unit-cell volumes are especially difficult for neutron crystallography owing to the low flux of neutron beamlines and the spatial overlap of reflections that large unit cells generate (Blakeley, 2011 ▸). The macromolecular neutron diffractometer (MaNDi) beamline at Oak Ridge National Laboratory (ORNL), commissioned in 2014, circumvented the challenge of this large unit cell by using time-of-flight Laue (*i.e.* multiwavelength) diffraction (Coates *et al.*, 2010 ▸, 2015 ▸). Time-of-flight information allows polychromatic diffraction patterns to be resolved into monochromatic slices, allowing the discrimination of the spatially overlapped reflections that are especially problematic for crystals with large unit cells (Langan *et al.*, 2008 ▸; Coates *et al.*, 2010 ▸). In conjunction with perdeuteration of MnSOD and moderate crystal volumes (>0.25 mm^3^), neutron diffraction using the MaNDi beamline was achieved to a resolution (2.14 Å) at which deuterium positions could be observed (Azadmanesh, Trickel, Weiss *et al.*, 2017 ▸).

After developing a pipeline for perdeuteration of MnSOD, large crystal growth and neutron diffraction to a resolution at which hydrogen positions can be visualized, we next endeavored to control the redox state of the manganese ion in this system. Visualizing a proton environment in which the manganese ions are either all trivalent or divalent (rather than the native mixed state) provides important snapshots of the proton-based catalytic mechanism (1), where differences in proton positions can then be used to track the path of proton transfers. In this work, several methods are provided for controlling the redox state of the active-site manganese ion within MnSOD crystals (*i.e.* after crystallization) for neutron crystallography. The redox-manipulation techniques described are intended to be applicable to other sorts of crystal treatments (neutron or X-ray) or other metalloprotein crystal systems.

## 2. Materials and methods   

### 2.1. Perdeuterated expression, purification and crystallization   

The details of the methods for the perdeuteration, expression, purification and crystallization of MnSOD have been described previously (Azadmanesh, Trickel, Weiss *et al.*, 2017 ▸).

### 2.2. Redox manipulation   

**Figure 1:**
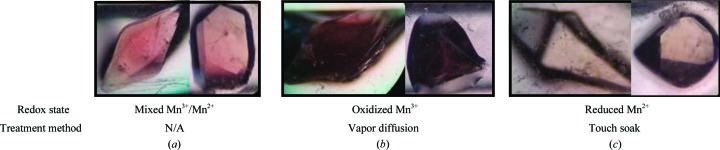
Representative images of (*a*) as-isolated, (*b*) oxidized and (*c*) reduced perdeuterated human MnSOD crystals. The oxidized crystals shown were obtained by the vapor-diffusion method (see §[Sec sec2]2) using deuterated substitute reservoir solutions supplemented with potassium permanganate within capillaries. The reduced crystals shown were obtained using the touch-soak method (see §[Sec sec2]2) using deuterated reservoir supplemented with sodium dithionite within capillaries. The images were taken with identical lighting and the crystal volumes varied from 0.2 to 0.5 mm^3^.

The reduction of MnSOD was achieved with (i) hydrogen peroxide, (ii) sodium ascorbate and (iii) sodium dithionite. Potassium permanganate was used as an oxidizing agent. These chemicals were supplemented into the deuterated substitute reservoir solution composed of 2.43 *M* deuterated potassium phosphate pH 7.4 (pD 7.8). The redox state of the manganese is detected by the intensity of the pink color of the crystals (Fig. 1 ▸). A deep pink color indicates manganese(III) ions and colorlessness indicates manganese(II) ions (Lah *et al.*, 1995 ▸; Lévêque *et al.*, 2001 ▸). Note that electroparamagnetic resonance and optical spectral studies verify the exclusive formation of manganese(III) by potassium permanganate oxidation and not manganese(IV) (Whittaker & Whittaker, 1991 ▸; Law *et al.*, 1998 ▸; Sheng *et al.*, 2012 ▸, 2013 ▸; Hunter *et al.*, 2015 ▸).

#### 2.2.1. Redox-agent screen   

Hydrogenated MnSOD was first crystallized in each well of a 24-well crystallization plate (a VDXm plate from Hampton Research) *via* hanging-drop vapor diffusion using identical crystallization conditions for each well: 1.8 *M* potassium phosphate pH 7.8. 1 µl each of reservoir solution and 23 mg ml^−1^ protein solution were used for the crystallization drop, with crystals growing to no larger than 0.05 mm^3^ (Azadmanesh, Trickel, Weiss *et al.*, 2017 ▸). Redox agent was then added to each reservoir solution in a gradient concentration across the whole plate along with concentrated potassium phosphate pH 7.8 to maintain the original molarity when necessary. The ‘new’ reservoir solution and the crystal were then allowed to vapor-diffuse with each other for one week. Persistence of the native light pink color (Fig. 1 ▸ *a*) was representative of too little redox agent, while cracked or deformed crystals were characteristic of too much. Concentrations of redox agent that were able to change the intensity of the pink color without visibly compromising the crystal integrity were used as a starting point to optimize the redox changes of larger crystals contained within a capillary.

#### 2.2.2. Redox manipulation of large crystals for neutron diffraction   

After mounting crystals in a capillary (the mounting method is described in detail in Azadmanesh, Trickel, Weiss *et al.*, 2017 ▸), three techniques were explored to control the redox state of the manganese ions in MnSOD crystals: vapor diffusion, a ‘touch soak’ and a full soak.

Firstly, the oxidation state of the manganese could be controlled solely through vapor diffusion by supplementing the redox reagent into the deuterated substitute reservoir solution intended to keep the crystal hydrated. The length of time over which the redox changes could be observed varied with the concentration of the agent and the distance between the solution and the crystal. This was the primary method of obtaining oxidized crystals and was achieved by allowing reservoir solutions supplemented with 6.4 m*M* potassium permanganate to vapor-diffuse with the sample.

Secondly, touch soaking was used when vapor diffusion was insufficient. This is achieved by cautiously using a pipet to move the redox agent-supplemented reservoir slug within the capillary to barely contact the crystal. The slug is then pipetted away when the redox change is complete, which is intended to occur within a time frame of seconds. This was the predominant method used to reduce crystals with hydrogen peroxide (0.25–1.00%).

Finally, a full soak of crystals in redox agent-supplemented deuterated substitute reservoir solution was performed while the sample was still within the capillary. Changes were observed within seconds, but soaks could be performed over several days to ensure a persistent shift in oxidation state. This was the primary method for obtaining reduced crystals and was achieved by soaking crystals in reservoir solution supplemented with 0.2 *M* sodium dithionite.

### 2.3. Neutron data collection   

Prior to data collection, the reservoir slugs in the capillaries bearing the crystal samples were replaced with fresh deuterated reservoir solution supplemented with redox agent. Neutron data were obtained from oxidized and reduced perdeuterated human MnSOD crystals (Table 1 ▸). Time-of-flight wavelength-resolved neutron Laue diffraction data (Langan *et al.*, 2008 ▸) were collected on the MaNDi instrument (Coates *et al.*, 2010 ▸, 2015 ▸) at the Spallation Neutron Source (SNS) using all neutrons with wavelengths between 2 and 4 Å. During the collection of each diffraction pattern the crystal was held stationary, and it was rotated by 20° between successive diffraction patterns. The diffraction data were reduced using the *Mantid* (Arnold *et al.*, 2014 ▸) software and were scaled and wavelength-normalized using the *LAUENORM* (Campbell, 1995 ▸) program from the *LAUEGEN* suite (Campbell *et al.*, 1998 ▸).

## 3. Results and discussion   

### 3.1. Redox manipulation   

To discover the optimal redox agent for manipulating the oxidation state of the manganese within the crystals, a variety of agents, concentrations and exposure methods were tested. Several redox agents and three manipulation methods were tested and their advantages and disadvantages are noted. Each agent and method had an appropriate use depending on the size and stability of the crystal as well as the susceptibility of the metal to redox changes (*i.e.* the redox potential) while maintaining an adequate diffraction quality. In the case of MnSOD, the redox changes of the active-site metal were detected by a change in the intensity of the pink color of the crystals (Fig. 1 ▸). A deep pink color is indicative of trivalent manganese ions, whereas colorless crystals represent divalent manganese ions (Lah *et al.*, 1995 ▸; Lévêque *et al.*, 2001 ▸).

#### 3.1.1. Redox-agent screen   

Four redox agents were screened using small crystals (<0.05 mm^3^) grown in a 24-well plate by supplementing the agent into the reservoir solution and allowing it to interact with the crystals *via* vapor diffusion.

Hydrogen peroxide is a well known oxidizing agent, but in the case of its interaction with the manganese of MnSOD it acts as a reducing agent when in excess by forcing the backwards reaction of the second half reaction in (1) (Hearn *et al.*, 2001 ▸). This is not feasible with copper- or iron-containing proteins as hydrogen peroxide imposes Fenton chemistry with these transition metals. Concentrations above 1% hydrogen peroxide abolished diffraction without visible alterations to the crystals other than their color, while 0.1% was able to turn these small crystals colorless.

Sodium ascorbate has been documented as a reducing agent for SODs in solution (Ranguelova *et al.*, 2012 ▸; Sheng *et al.*, 2012 ▸). Signs of reduction were not detected with concentrations of up to 0.85 *M* after one week of vapor diffusion or when soaking the crystals overnight. After one month, color changes were visible with concentrations of 0.85 *M* solely using vapor diffusion.

Sodium dithionite has been applied in earlier X-ray crystallographic studies of SOD using soaking methods (Lah *et al.*, 1995 ▸; Lévêque *et al.*, 2001 ▸) and has been shown to influence the absorption spectra of the MnSOD chromophore (Sheng *et al.*, 2012 ▸). In the vapor-diffusion screen, concentrations of greater than 0.3 *M* sporadically led to the growth of salt crystals within the reservoir, on the crystals or within the crystals. For the small crystals of this screening method, 0.2 *M* was sufficient to turn the crystals colorless after 1 d.

Potassium permanganate has been shown to increase the absorption spectra of the oxidized MnSOD chromophore (Sheng *et al.*, 2012 ▸). It was not observed to have ‘side effects’ on the crystals when using concentrations of up to 7 m*M*, which easily increased the pink intensity of the samples. Higher concentrations were not used owing to concern over unwanted long-term effects.

While the screening described above determined that redox manipulation of small MnSOD crystals was possible, the techniques needed to be translated to larger crystals (>0.2 mm^3^) to perform neutron diffraction. The suitable redox-agent concentrations discovered using the small-scale screen were used as a starting point to achieve redox manipulation of larger MnSOD crystals within capillaries. Finding the optimal concentrations for larger crystals in capillaries was more difficult owing to the large variability in crystal sizes, as the amount of redox agent needed to change the redox state of the samples is proportional to the crystal volume. The distance between the reservoir slug and the crystal within a capillary also is a determinant of the potency of redox influence on the sample using vapor diffusion. Below, redox manipulation *via* vapor diffusion for neutron crystallography is addressed along with alternative methods for controlling the oxidation state of the active-site manganese ion.

#### 3.1.2. Redox manipulation *via* vapor diffusion for neutron crystallography   

**Figure 2:**
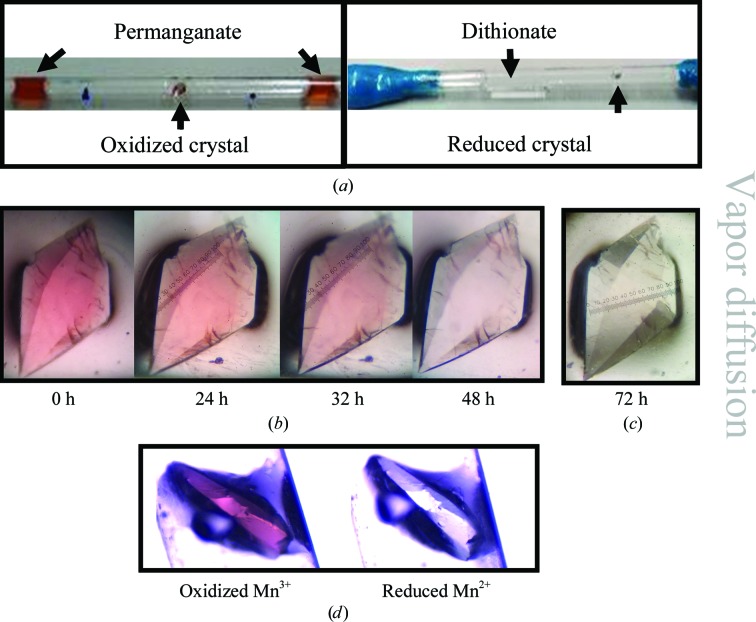
Redox manipulation of perdeuterated human MnSOD crystals *via* vapor diffusion within capillaries. Cracks were present prior to manipulation. (*a*) Images of crystals within capillaries along with deuterated reservoir supplemented with either potassium permanganate for oxidation or dithionite for reduction. (*b*) Time course of crystal reduction using reservoir solutions supplemented with 1% hydrogen peroxide. The purple/pink background is from the microscope polarizer with its setting maintained between images, while the lighting was consistent between the images in this panel. (*c*) Complete reduction by hydrogen peroxide is observed by the absence of pink color that is achieved 72 h after supplementation with hydrogen peroxide. The image was taken with alternative lighting compared with (*b*) and without the polarizer to better observe the colorlessness. Diffraction quality was maintained during this time course (data not shown). (*d*) The same crystal was oxidized by supplementation with potassium permanganate and reduced with sodium dithionite. The purple background is from the microscope polarizer with its setting maintained between images. The lighting was kept consistent between the images.

Vapor diffusion within a capillary is considered to be the least invasive method of redox manipulation, with the minimal amount of risk to crystal integrity as the crystal is not touched after it has been mounted in a capillary. This technique involves simply adding the redox reagent to the deuterated substitute reservoir slugs in the capillary (Fig. 2 ▸ *a*), allowing vapor diffusion of both D_2_O and the redox agent with the crystal. The speed of the redox change is proportional to the concentration of the redox agent in the reservoir slug and the distances of the slug(s) from the crystal. In general, a balance between these three elements should be considered. Redox and color changes can take between days and weeks depending on the parameters used. High amounts of a redox compound or close proximity of the slug and crystal increases the susceptibility of the sample to side effects. These include cracking of the crystal, salt or protein aggregates nucleating within the protein crystal and/or deteriorating diffraction quality. Some crystals were more susceptible to these side effects than others when the conditions were kept identical. This was probably owing to differences in the intrinsic quality of the crystals. The capillaries can be opened to replace the supplemented reservoir slugs with fresh ones every few days to speed up the process, but this also increases the likelihood of damaging the crystals owing to handling. While vapor diffusion has the potential to be one of the least invasive tech­niques, one needs to compromise between time and risk when manipulating the samples.

The crystals were oxidized primarily through vapor diffusion owing to the lack of apparent side effects from using 6.4 m*M* potassium permanganate, permitting liberal volumes of permanganate and close proximity between the slugs and the crystal (∼3 cm). Potassium permanganate increased the pink color of the crystals over the course of one week (Figs. 1 ▸ *b* and 2 ▸ *a*). During the vapor-diffusion process, the pink color of the permanganate solution decreased, indicating decay of the oxidizing agent. Upon this observation, the capillaries were opened and the reservoir slugs were replaced with slugs containing ‘fresh’ permanganate. After several replacements, the pink color of the solution stabilized within the sealed capillary and the crystals retained the deep pink intensity over the course of several months, which is likely to be a consequence of saturation of the redox reaction. Subsequent neutron diffraction utilizing this method was achieved on MaNDi to 2.14 Å resolution (Table 1 ▸).

Reducing the manganese within the crystals using vapor diffusion was achieved, but not without difficulties. Hydrogen peroxide concentrations ranging between 0.25 and 1.00% were able to remove the pink intensity of the crystals in 1–3 d (Figs. 2 ▸ *b* and 2 ▸ *c*), representative of divalent manganese ions within the sample, but at a consequence. The diffraction quality of these crystals seemed to be unaffected for approximately two weeks, but was rapidly abolished afterwards with no apparent changes in the visual quality of the samples. This may indicate saturation of the redox reaction between hydrogen peroxide and manganese, and funneling of hydrogen peroxide towards decomposing the protein by oxidizing reactions. The diffraction quality of a crystal could be compromised before the experiment is completed, given the longer data-collection times needed with neutrons. Even with this deleterious side effect, the use of hydrogen peroxide as a reducing agent is feasible if data collection is performed within an adequate time frame, such as with X-rays. Alternatively, hydrogen-peroxide-soaked crystals could be cryotrapped for neutron data collection.

#### 3.1.3. Redox manipulation *via* a ‘touch soak’ for neutron crystallography   

**Figure 3:**
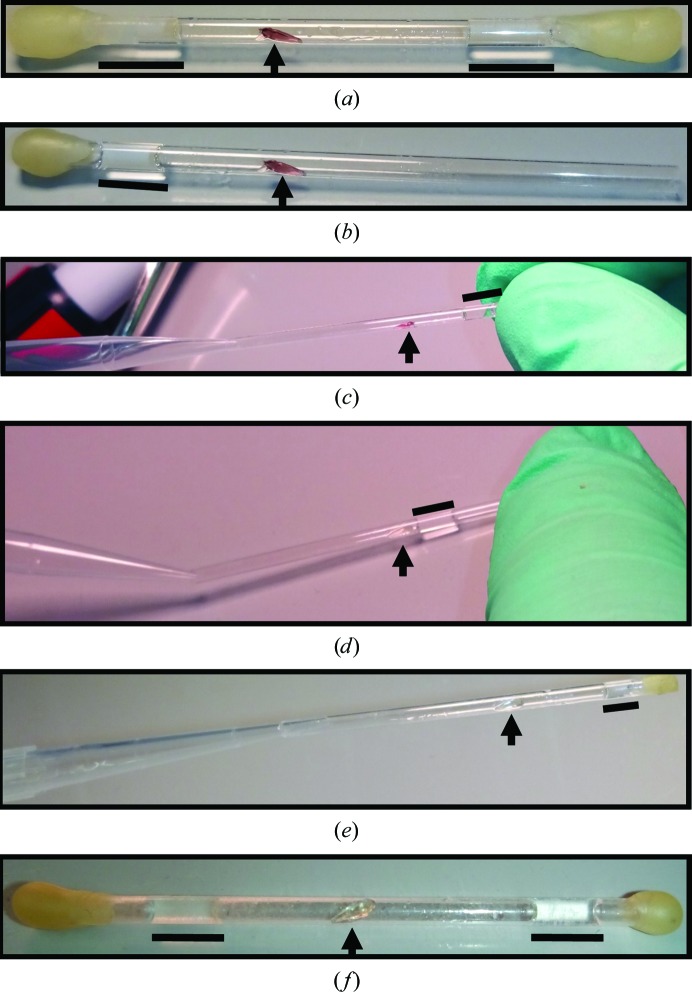
Procedure for the ‘touch-soak’ method for reduction of perdeuterated MnSOD crystals. Arrows indicate the position of the crystal, while black lines indicate the positions of deuterated reservoir slugs supplemented with dithionite. (*a*) An MnSOD crystal flanked by dithionite-containing slugs within a capillary sealed with melted wax. (*b*) At one end, the wax is peeled off and the slug is removed. (*c*, *d*) Using a pipet, the remaining slug is gently moved using negative pressure to make minimal contact with the crystal. The redox change of the crystal is noticeable within minutes. In some cases, only contact with the mother liquor is needed. This can be performed with or without the other end of the capillary sealed. If sealed, the slug will jump back to its original position once the negative pressure of the pipet is removed; otherwise, positive pressure with a pipet is applied to move the slug back to its original position. (*e*, *f*) Another slug supplemented with dithionite is added using a pipet attached to a gel-loading pipet tip and the ends are sealed with melted wax.

The advantage of vapor diffusion is the ability to chemically treat the crystals without direct contact, but its appeal diminishes when the higher concentrations of reagent used in the process compromise the samples, such as with sodium dithionite. Another means to treat the samples is to lessen the concentration of the chemical agent in the reservoir slug and allow very little contact with the crystal, minimizing any potential damage to the sample. In the case of MnSOD, 0.2 *M* dithionite was able to reduce crystals using this approach, a concentration where aggregate growth was significantly less than at 0.3 *M*. This is achieved by cautiously using a pipet to move the redox agent-supplemented reservoir slug within the capillary to barely create contact with the crystal (Fig. 3 ▸). The slug is then pipetted away when the redox change is complete. The effects of the dithionite on the samples were noticeable within minutes, totally abolishing the intensity of the pink color of the crystals. In some cases, only contact with the mother liquor surrounding the crystal was needed. After redox treatment, the dithionite-supplemented reservoir slugs were replaced weekly to maintain a reducing environment within the capillary, as dithionite decomposes in solution. This method also ensures more extensive deuterium exchange between the crystal and the deuterated substitute reservoir than the vapor-diffusion method. Neutron diffraction to a resolution at which hydrogen positions could be observed was possible (Table 1 ▸). The advantages of this ‘touch-soak’ method include the use of a lower concentration of agent and rapid effects, while difficulties can arise from the increased mosaicity and the precision handling required.

#### 3.1.4. Redox manipulation *via* a full soak for neutron crystallography   

**Figure 4:**
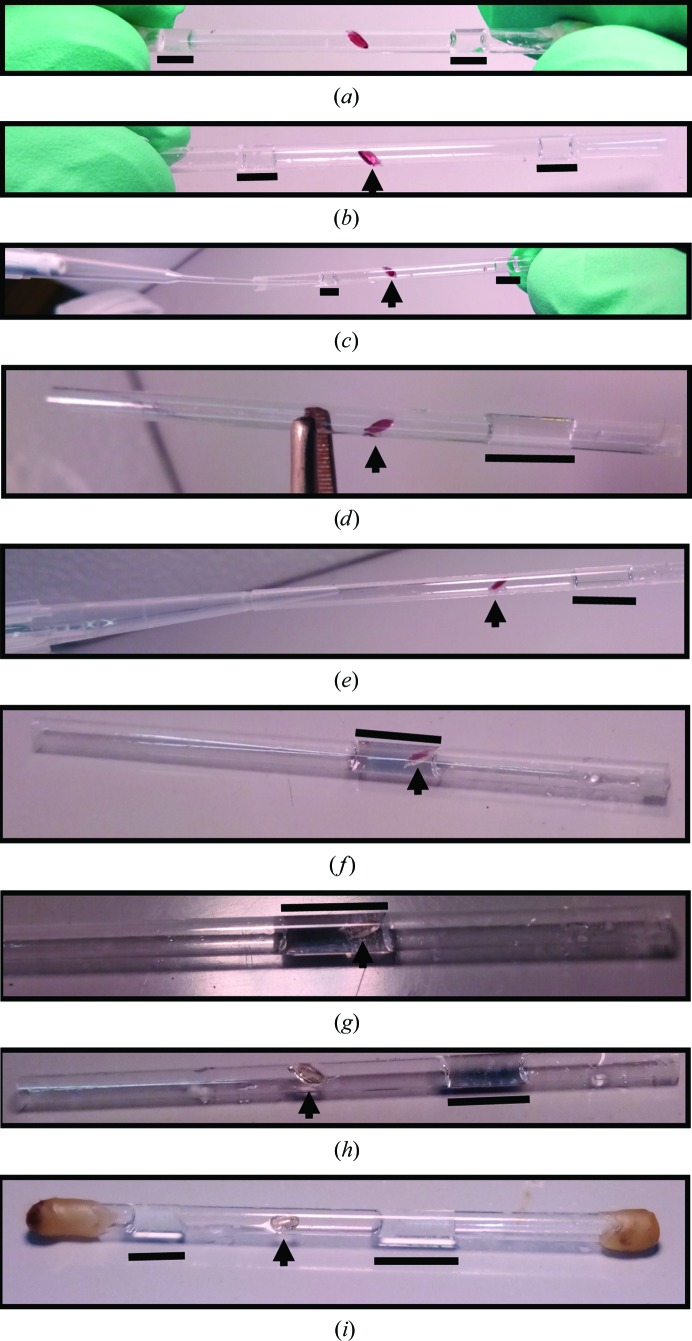
Procedure for reducing perdeuterated MnSOD crystals by a full soak within a capillary. Arrows indicate the position of the crystal, while black lines indicate the positions of reservoir slugs supplemented with dithionite. (*a*) An MnSOD crystal flanked by dithionite-containing slugs within a capillary heat-sealed using wax. (*b*) Both ends of the capillary are opened by peeling off the wax (without heat). (*c*, *d*) One of the slugs is removed using a pipet attached to a gel-loading pipet tip. (*e*, *f*) Using a pipet, negative pressure is applied to engulf the crystal in the slug solution. (*f*) shows a crystal transitioning between redox states ∼20 s after initiation of the soak, while (*g*) depicts the full transition after 1 min. (*h*) The soaking solution is subsequently pipetted off or dried using wicks. During this process, it should be ensured that a slug is present within the capillary to ensure crystal hydration. (*i*) Another slug is added and the capillary is resealed.

**Figure 5:**
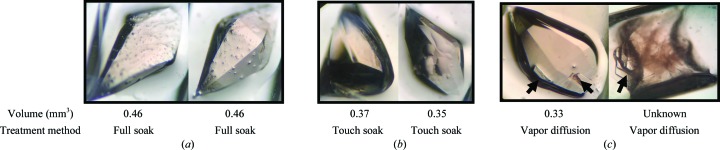
Effects of crystal size and dithionite-treatment method on MnSOD crystal integrity. (*a*) Crystals that were soaked in a deuterated substitute reservoir solution supplemented with 0.2 *M* dithionite for 3 d. The small specks appeared one week after removal of the soaking solution. These could be salt or protein aggregates. A full neutron data set was collected from the crystal shown in the left image owing to its size and lower mosaicity (judged by X-ray diffraction) compared with the other samples. (*b*) Crystals treated with dithionite by the touch-soak method using the same solution as in (*a*). The larger crystals in (*a*) re-oxidized from the reduced state when using the touch-soak method, which can be attributed to their ∼25% larger volumes compared with those in (*b*). (*c*) Images of crystals treated with 0.3 *M* dithionite by vapor diffusion. Arrows indicate aggregates on or within the crystals. The left image depicts a minor detriment from aggregates to the protein crystal quality, whereas the right image shows aggregate growth within the sample that significantly affects the quality of the protein crystal.

MnSOD crystals that were larger in size, >0.4 mm^3^, were resistant to redox changes through both vapor diffusion and touch soaking. The redox state of these crystals would revert to the native mixed state over the course of several days. This is a consequence of the increased amounts of the metalloprotein contributing to the crystal size, requiring larger amounts of redox agent to shift the oxidation state of the crystal. In the case of sodium dithionite, increased concentrations (>0.3 *M*) for the vapor-diffusion or touch-soak methods were not feasible owing to the worsening of un­favorable effects on the samples. To keep the concentrations of the agent low and still achieve shifts in the oxidation state of the crystals without reversion, full soaks were performed within the capillaries. These consisted of gently engulfing the crystals with deuterated substitute reservoir slugs supplemented with 0.2 *M* sodium dithionite (Fig. 4 ▸). Some samples cracked when drowned in redox solution; these were usually poor-quality crystals, but one must nonetheless consider this risk. The pink intensity of the crystals faded within 1 min of soaking, after which the solution can be pipetted off and the crystal laid dry. Some crystals could be soaked in the dithionite solution for weeks and still maintain adequate diffraction quality. Once the crystals had been laid dry, dithionite-supplemented (0.2 *M*) reservoir slugs were placed in the capillary to maintain a reducing environment *via* vapor diffusion. One week after removal of the soaking solution, small aggregates were observed on the protein crystal (Fig. 5 ▸ *a*). These aggregates did not grow larger and did not seem to exacerbate the diffraction quality. Reversion of the oxidation state was not observed after a month. Full soaks are advantageous in their ability to ensure a redox shift of samples as well as promoting rapid deuterium exchange for neutron crystallography, although the crystals are susceptible to cracking and may exhibit increased mosaicity.

### 3.2. Optimizing treatment conditions and determining which technique to use   

Discovering the optimal conditions to treat large crystals intended for room-temperature neutron diffraction has the potential to be a lengthy process. A brief workflow has been outlined to minimize the time required to find the appropriate treatment conditions for each of the three techniques. This proposed process assumes that the effects of treatment can be observed without diffraction, such as by eye or from crystal absorption spectra, and that screening will be performed on large hydrogenated crystals.

(i) Find the minimum concentration of treatment agent (supplemented into the substitute reservoir solution) needed to observe the desired effects within minutes on samples upon a full soak. The rationale is that the concentration of treatment agent with full soaks is the least variant with crystal size as opposed to touch soaks or vapor diffusion. In addition, the change within a short time frame is a rough indicator of whether the desired effects can be maintained with vapor diffusion in a sealed capillary after removal of the soaking solution. If the redox changes are immediate then relapse is less likely.
(ii) A compatible concentration of treatment agent for touch soaking is likely to be very similar to the concentration used for full soaks. Similar to the full soak, touch soaking should yield the desired effects within minutes. Touch soaking is more susceptible to reversion of the desired state owing to its less rigorous nature and may require an increased concentration of agent compared with that in the full soak. The size of the crystals may also be another determinant, where smaller crystals may require lesser amounts of the treatment agent to obtain the desired effects.
(iii) Vapor diffusion will require a higher concentration of treatment agent to observe changes within days/weeks compared with the touch-soak and full-soak methods to compensate for its less invasive nature. The concentrations of agent discovered from full-soak and touch-soak screens serves as a rough starting point for vapor-diffusion screens, with increases of up to 50% being needed. An alternative way to increase the speed of crystal changes from treatment is to decrease the amount of distance between the agent-containing slug and the sample, as well as periodically replacing the slug with freshly made agent-containing solution.

### 3.3. Feasibility for neutron crystallography   

One intent of this work was to gauge the feasibility of collecting neutron data from redox-treated, large, perdeuterated MnSOD crystals to a resolution where the positions of H atoms are observed. The possibility of this was unclear given the large unit-cell dimensions of the MnSOD crystal form, which crowds diffraction spots, and the tendency for chemical crystal treatments to decrease diffraction quality, particularly through exacerbating spot crowding. Too much spot overlap ultimately reduces the resolution of the data. Neutron data sets were collected from crystals in which manganese ions had been manipulated into the trivalent state (‘oxidized’) or the divalent state (‘reduced’) (Table 1 ▸). The higher *R*_merge_ values than would be expected for monochromatic X-ray data are comparable with other Laue data sets collected using neutron spallation sources (Chen *et al.*, 2012 ▸).

#### 3.3.1. Neutron data collection from perdeuterated MnSOD crystals treated with redox agents   

Collecting data from oxidized perdeuterated MnSOD crystals did not involve any additional difficulties to those already inherent to neutron crystallography. The crystal from which data was collected was 0.3 mm^3^ in size and was oxidized by vapor diffusion using potassium permanganate implemented into the deuterated substitute reservoir slug (Fig. 2 ▸ *a*, left). Neutron data were collected and processed to 2.14 Å resolution and are the highest resolution data obtained for this crystal system.

For data collection from a reduced sample, a crystal of 0.46 mm^3^ in size soaked in dithionite for 3 d was chosen (Fig. 5 ▸ *a*, left). One week after removal of the soaking solution, small aggregates were observed on the protein crystal. These aggregates did not grow larger and did not seem to exacerbate the diffraction quality. Before data collection, the slugs within the capillary containing the sample were replaced by fresh dithionite-supplemented deuterated reservoir solution. After collecting data for 16 d, the crystal maintained its redox state (*i.e.* colorlessness). The diffraction data were strong, with a diffraction limit of ∼2.0 Å resolution, which was attributed to the size and perdeuterated qualities of the sample. Despite the extent of diffraction, the quality of the data was hampered by spot overlap at higher resolutions. This is inherently an issue because of the unit-cell dimensions, but was further exacerbated by the increased mosaicity from dithionite treatment, which increased the size of the Bragg reflections. From the corresponding X-ray data, the mosaicity of this crystal was 0.49° compared with the value of 0.26° for the oxidized crystal. Consequently, the resolution had to be cut to 2.30 Å during processing, which is still sufficient to identify hydrogen positions.

As a comparison, our previous work details neutron data collection from a perdeuterated MnSOD crystal that was not redox-manipulated (Azadmanesh, Trickel, Weiss *et al.*, 2017 ▸). This crystal was 0.26 mm^3^ in volume and data were collected and processed to 2.30 Å resolution. While this untreated crystal was of smaller size compared with its treated counter­parts, it is apparent that the redox-manipulation methods discussed here do not significantly impair the resolution to which neutron data can be collected and processed.

## 4. Conclusions   

### 4.1. Mechanism of the interaction between redox agents and MnSOD crystals   

Both the size and the charge of the redox agents allow an interaction with the active-site manganese ion of MnSOD. The ∼5 Å opening of the active-site channel of MnSOD is large enough to allow both permanganate and dithionite to enter and interact with the manganese (Weinrach *et al.*, 1992 ▸; Bühl, 2002 ▸; Azadmanesh, Trickel & Borgstahl, 2017 ▸). Both of these molecules are negatively charged and are likely to be guided to the active site by the same positive electrostatic surfaces of MnSOD that promote the productive diffusion of anionic superoxide for its catalysis (Azadmanesh, Trickel & Borgstahl, 2017 ▸). For the negatively charged ascorbate, redox changes were observed at a much slower rate. This could be attributed to its bulkier size compared with the other agents tested. The size of ascorbate only permits it to enter the active site in specific orientations. Nevertheless, a near-direct interaction between redox agents and the active-site manganese ion is possible.

The redox state can be influenced without any direct contact between the agent and the sample *via* vapor diffusion, indicating that permanganate, dithionite, hydrogen peroxide and ascorbate can all diffuse through the capillary as a gas. The means by which this occurs is unclear. Diffusive water molecules could hydrate the redox agents, and the evaporative hydration complex may be capable of traveling through the capillary. Nevertheless, redox agents that do not make contact with crystals within capillaries do exert observable effects (Fig. 2 ▸).

### 4.2. Hydrogen peroxide as a reducing agent and a substrate   

MnSOD is unique compared with FeSOD and CuZnSOD in that the active-site metal can be reduced by excessive amounts of hydrogen peroxide. MnSOD exhibits reversible product inhibition by hydrogen peroxide, which is not observed for the other SODs owing to the susceptibility of iron and copper to Fenton chemistry. Addition of copious amounts of hydrogen peroxide to MnSOD instigates the formation of a product-inhibited complex, which can only form when manganese is in the trivalent state (Srnec *et al.*, 2009 ▸). Subsequently, the presence of the Mn^3+^-peroxo complex decays in conjunction with the formation of Mn^2+^SOD. This phenomenon has been explained by Hearn *et al.* (2001 ▸): ample amounts of hydrogen peroxide first bind as the inhibited complex and then force the backwards reaction to produce superoxide in tandem with an electron reduction of Mn^3+^ to Mn^2+^. The generated superoxide can then interact with an Mn^3+^ ion to instigate the forward reaction with an electron reduction to yield diatomic oxygen (O_2_) and Mn^2+^. Hydrogen peroxide is thought to be incapable of binding to Mn^2+^ owing to steric hindrance by the water molecule ligated to the manganese (Srnec *et al.*, 2009 ▸). The water molecule is deprotonated to hydroxide in the Mn^3+^ state, which provides space for hydrogen peroxide to bind. The generated superoxide is capable of interacting with Mn^2+^ to oxidize the cation to a trivalent (Mn^3+^) state, but the excessive amounts of hydrogen peroxide and its cascade of reactions yield Mn^2+^ as the dominant species.

The attribute of hydrogen peroxide acting both as an inhibitor and a reducing agent explains why hydrogen peroxide-soaked structures of MnSOD may not reveal peroxide at the active site. This is exemplified by two hydrogen peroxide-soaked *Escherichia coli* MnSOD structures. One soaked with 0.4% hydrogen peroxide did not reveal peroxide at the active site (PDB entry 1ixb; B. F. Anderson, R. A. Edwards, W. M. Whittaker, J. W. Whittaker, E. N. Baker & G. B. Jameson, unpublished work), while another soaked with 0.008% hydrogen peroxide did (PDB entry 3k9s; Porta *et al.*, 2010 ▸). The former contains divalent manganese ions based on quantum-mechanical/molecular-mechanics (QM/MM) calculations (Rulíšek & Ryde, 2006 ▸), whereas the redox state of the latter cannot be discriminated owing to the peroxide being observed with partial occupancy. The amounts of hydrogen peroxide used in these soaks may determine whether a backwards reaction is forced or whether only binding to form a product-inhibited complex occurs.

The slow decay in crystal diffraction quality as a result of hydrogen peroxide treatment may be a consequence of oxidative damage. Hydrogen peroxide is known to react with the amino acids cysteine, methionine, lysine, histidine and glycine (Finnegan *et al.*, 2010 ▸). Flash-cooling to cryogenic temperatures has been shown to circumvent this deleterious effect (Rulíšek & Ryde, 2006 ▸; Porta *et al.*, 2010 ▸) for X-ray crystallography. Cryocooling is notoriously difficult for the large crystals needed for neutron crystallography, but is possible (Casadei *et al.*, 2014 ▸; Li *et al.*, 2017 ▸). Cryogenic neutron crystallography has the potential to be amenable for large MnSOD crystals owing to the built-in Oxford Cryo­systems Cobra cryostream at the MaNDi beamline, which is capable of temperatures between 80 and 200 K (Coates *et al.*, 2014 ▸, 2015 ▸).

### 4.3. Future directions   

Neutron data are normally refined with an X-ray data set from the same crystal or from another mimicking the growth and treatment conditions of the original (Afonine *et al.*, 2010 ▸). This is because neutron and X-ray data result in different but complementary information. X-ray structures are typically of higher resolution, which can be used to derive restraints for refining neutron data. However, this poses an issue for crystal structures that are intended to be fully oxidized, as X-ray exposure reduces the metal of metalloproteins while neutrons do not (Carugo & Djinović Carugo, 2005 ▸). To circumvent this issue, restraints/data from the X-ray data set need to be omitted for the area of the molecule that will be influenced by redox changes. For example, the active-site metal ion and its ligated amino acids as well as any neighboring residues should not incorporate X-ray data during refinement. An alternative for restraining these active-site molecules is to input custom restraints derived from QM/MM or density-function theory (DFT) calculations. In the case of MnSOD, DFT calculations for the oxidized human form have been published (Abreu *et al.*, 2005 ▸).

## Figures and Tables

**Table 1:** Data-collection statistics

Oxidation state of Mn	Oxidized (3+)	Reduced (2+)
Diffraction source	MaNDi	MaNDi
Size (mm^3^)	0.30	0.46
Wavelength(s) (Å)	2–4	2–4
Temperature (K)	293	293
Detector(s)	40 SNS Anger cameras	40 SNS Anger cameras
Crystal-to-detector distance (mm)	450	450
Rotation range per image (°)	0	0
No. of images collected	6	8
Total rotation range (°)	120	160
Exposure time per image (h)	48	48
Space group	*P*6_1_22	*P*6_1_22
*a*, *b*, *c* (Å)	81.4, 81.4, 242.3	81.4, 81.4, 242.3
α, β, γ (°)	90, 90, 120	90, 90, 120
Resolution range (Å)	15.27–2.14 (2.22–2.14)	15.67–2.30 (2.38–2.30)
Total No. of reflections	68993	77229
No. of unique reflections	21386	20454
Completeness (%)	80.0 (69.3)	93.2 (93.6)
Multiplicity	3.23 (1.94)	3.78 (3.46)
〈*I*/σ(*I*)〉	4.50 (2.70)	5.7 (4.10)
*R*_merge_ (%)	21.7 (28.2)	22.0 (22.8)
*R*_p.i.m._ (%)	10.1 (21.4)	11.4 (15.7)
